# 816. Demographic Analysis of a Randomized Controlled Trial Comparing Outcomes of Dermal Matrices in Burn Injuries

**DOI:** 10.1093/jbcr/irag033.255

**Published:** 2026-04-06

**Authors:** Jeffrey E Carter, Tina L Palmieri, Sigrid Blome-Eberwein, John Cockwill

**Affiliations:** University Medical Center Burn Center, Louisiana State University Health Sciences Center New Orleans, New Orleans, LA; University of California Davis Health, Shriners Children’s - Northern California, Sacramento, CA; Lehigh Valley Health Network Regional Burn Center, Allentown, PA; PolyNovo Biomaterials, Clearwater, FL

## Abstract

**Introduction:**

Severe burn injuries often require temporizing strategies before definitive wound closure. Advanced Dermal Matrices (ADMs) are designed to integrate into the wound bed and support subsequent split-thickness skin grafting (SSG). This pivotal, randomized, controlled trial (RCT) evaluates the safety and effectiveness of a novel, synthetic, biodegradable dermal matrix* compared to standard of care (SOC) treatments in patients with deep dermal and full-thickness thermal burns to determine the safety and efficacy of the novel ADM.

**Methods:**

This multicenter, IDE, RCT was sponsored by BARDA and enrolled adult patients (18–75 years) with 3–60% TBSA burns. Subjects were randomized 2:1 to receive the novel, synthetic, biodegradable dermal matrix or SOC (e.g., an ADM containing cross-linked bovine collagen and chondroitin-6-sulfate or cadaveric allograft) before SSG. The primary effectiveness endpoint is the total percent wound closure calculated for each randomized subject based on an Independent Panel assessment of study lesions at 4 weeks after split-thickness skin grafting and serves as the dependent variable. The primary safety endpoint is a composite of device (BTM group)-/treatment (SOC group)-related mortality, skin graft loss requiring re-operation and device (BTM group)-/treatment (SOC group)-related AEs requiring re-operation. In this trial the subject served as the sampling unit for this composite endpoint; individual elements of this endpoint, such as skin graft loss, infection, and matrix removal, and is tabulated based on each treated burn site. Twenty-two sites in the USA and 3 in India participated.

**Results:**

127 subjects were enrolled and 120 randomized and treated with the first enrollment on 9-20-2021, and the final enrollment on 08-19-2024. Demographic Data is in the attached table.

**Conclusions:**

The CP-003 study is among the largest RCT performed to date comparing the outcomes of dermal matrices in deep second- and third-degree burns. The demographic data demonstrate excellent randomization between the novel, synthetic, biodegradable dermal matrix and SOC. Efficacy and safety results are being finalized for submission to the FDA as part of a PMA submission package and will be shared once the PMA review is complete.

**Applicability of Research to Practice:**

Safety and clinical efficacy of a novel, synthetic, biodegradable advanced dermal matrix that may promote faster healing and have improved cosmetic outcomes in severe burn injuries.

**Funding for the study:**

The Biomedical Advanced Research & Development Agency (BARDA).

